# The relationship between moral courage, team work, and safe nursing care in clinical nurses: a multicenter cross-sectional study in Iran

**DOI:** 10.1186/s12912-024-02097-3

**Published:** 2024-06-19

**Authors:** Zahra Azizi, Mohammad Mehdi Naghizadeh, Mostafa Bijani

**Affiliations:** 1 Student Research Committee, School of Nursing, Fasa University of Medical Sciences, Fasa, Iran; 2 Department of Epidemiology and Biostatistics, Fasa University of Medical Sciences, Fasa, Iran; 3 Department of Medical Surgical Nursing, School of Nursing, Fasa University of Medical Sciences, Fasa, 81936-13119 Iran

**Keywords:** Moral courage, Teamwork, Nurses, Safe care, Quality of healthcare

## Abstract

**Background:**

Moral courage and team work are the most important aspects of professional competence in clinical nurses; nurses with moral courage and team work are thought to be able to deliver safe nursing care to patients. The present study aimed to investigate whether moral courage and teamwork correlate with safe nursing care among clinical nurses.

**Methods:**

This descriptive cross-sectional multicenter study was carried out from December 2023 to February 2024. A total of 375 nurses who were practicing in four hospitals in the south of Iran were enrolled in this study using convenience sampling. The data collection tools used consisted of a demographics survey, Moral Courage Questionnaire (MCQ), Team STEPPS Team Perception Questionnaire (T-TPQ), and the Assessment of Safe Nursing Care Questionnaire (ASNCQ). The data were analyzed using descriptive statistics, t-test, chi-square, multiple regression analysis, and Pearson’s correlation coefficient. SPSS version 22 was used to analyze the data.

**Results:**

The participants’ mean age was 32.66 ± 6.63 years, and their work experience was 8.56 ± 6.22 years. The total mean scores for moral courage, teamwork, and safe care were 422.37 ± 52.92, 144.09 ± 18.43, 315.84 ± 41.95, respectively. A statistically significant positive correlation was found between teamwork and safe care (*r* = 0.57, *p* < 0.001), teamwork and moral courage (*r* = 0.49, *p* = 0.002), and moral courage and safe nursing care (*r* = 0.59 *p* < 0.001). According to the results, work experience, moral courage, and teamwork explained 44.4% of the variance in safe nursing care (R2 = 0.44, *p* < 0.001).

**Conclusion:**

The results indicated that the moral courage and teamwork of nurses were positively and significantly correlated with the participants’ safe nursing care. Accordingly, since moral courage and teamwork are the qualities that can contribute to improving the quality of care and ensuring safe nursing care, it is recommended that nursing managers pay special attention to these factors.

## Introduction

Healthcare systems are becoming more intricate and specialized, while patients' needs and health issues have become more complicated [1]. All healthcare providers, especially nurses as a large and main group of them, must adhere to all aspects of patient safety to provide safe care [2]. Safe nursing care is defined as the use of nurses' knowledge and skills to provide high-quality care so that the risk of harm is reduced for patients along nursing care [3]. On the other hand, nurses, due to their professional status and their role consistently face ethical challenges and issues that are closely linked to the quality of patient care. They need to have moral courage to refrain from unethical choices and choose the best options consistent with ethical principles as well as widely accepted values of the healthcare system [4]. Moral courage is recognized as a core value in the nursing profession; this value, along with two others, such as love and respect, was introduced by the International Nurses Association [5]. Moral courage is defined as an essential trait that enables nurses to make ethical decisions and navigate moral challenges effectively specially when protecting the patient's privacy and announcing bad news [6]. However, sometimes nurses may choose not to act morally due to the fear of losing their job, negative responses from colleagues, and decreased pay. This can cause feelings of moral distress, depression, guilt, anger, powerlessness, and feeling of worthlessness [7].

Further, moral courage is an important virtue in nursing which promotes both personal and professional growth [6]. Years ago, Florence Nightingale regarded moral courage as a crucial aspect of nursing care. Furthermore, researchers have underscored the impact of nurses' moral courage on enhancing the quality of care by emphasizing the importance of advocacy [8]. According to Kashani et al., the enhancement of moral courage can improve the quality of healthcare. Nurses with moral courage are always at their patients’ side, view their patients as human beings with different needs, empathize with them, and care about their interactions [9].

The studies conducted in Iran have reported varying levels of moral courage among nurses. In this regard, the results of studies by Khatiban et al. and Mohadeseh et al. have reported high levels of moral courage among nurses [10, 11]. However, based on the findings of Abdollahi, et al. as well as Aminizadeh et al., the level of moral courage among nurses has been reported as moderate to low. Consequently, there is a perceived need for further research on moral courage and its associated factors [12, 13]. The findings of Arablarimi, et al.'s study in Iran indicate that factors such as organizational climate, organizational support, work experience, psychological empowerment, ethical leadership and ethics education significantly influence the moral courage of nurses [14]. The findings of the study conducted by Rakhshan, et al., aimed at investigating nurses' perspectives on the barriers influencing moral courage in Iran, demonstrated that factors such as lack of job motivation and absence of organizational support, lack of job interest, low self-confidence, fear of outcomes, fear of rejection, physician paternalism, defective communication, and lack of powerful role models constitute the most significant barriers to moral courage among nursing professionals [15].

Teamwork in healthcare is referred to as a ‘dynamic process involving two or more health professionals with complementary backgrounds and skills, sharing common health goals and exercising concerted physical and mental effort in assessing, planning, or evaluating patient care’ Thus, for delivery of safe care, besides moral courage, healthcare providers should have sufficient knowledge about the significance of team working [16].

In addition to organizational efficiency, the teamwork skills of nurses are key indicators of their work performance and have a positive effect on their safe as well as high-quality care [17]. From the perspective of nurses in southern Turkey, teamwork and collaboration among healthcare professionals have important outcomes including both patient and staff satisfaction, cost efficiency, and the improvement of healthcare services [18]. According to Nobahar, et al., a higher level of teamwork reduced missed nursing care among ICU nurses [19]. Also, Kalisch et al. indicated that the nature and extent of missed nursing care were influenced by the level of nursing teamwork [20]. According to Gagnon, the shift towards team-based healthcare delivery has heightened the emphasis on teamwork. Effective teamwork can significantly enhance the quality of patient care. Accordingly, nursing managers must actively cultivate this essential skill among their staff. The absence of teamwork competency leads to task overlap, duplication of effort, as well as a waste of time and resources, ultimately negatively impacting the quality of nursing care [21]. The findings of Bahrami's et al. study, conducted with the aim of evaluating the competence of emergency nurses in Iran in performing teamwork, revealed that nurses lacking the necessary skills for effective teamwork are unable to adequately manage and organize the emergency department during critical situations when the emergency room becomes excessively crowded. Thus, they experience anxiety, confusion, and precipitance, which adversely impacts their professional performance. Therefore, there is a pressing need for further research across various departments concerning teamwork among nurses [22].

Various factors such as the prevalence of various diseases and the complexity of care in the health system have caused nurses to encounter caring challenges and ethical conflicts, so that they need teamwork skills and moral courage to provide safe nursing care. It is important to assess the nurses’ courage, teamwork, and safe care in identifying the areas that need to be improved, determining educational needs, as well as ensuring optimal care delivery and will lead to professional development. The review of the literature revealed that few studies have tried to explore the relationship between moral courage, teamwork, and safe nursing care among clinical nurses. Furthermore, no study has investigated the interaction of these factors, which needs to be specified; this can make our study the starting point for indicating the exact relationship between them. When there is a relationship among teamwork, moral courage, and safe nursing care among nurses, emphasis on the significance of planning to improve teamwork can help the health systems achieve wider goals in safe nursing care delivery. Given the importance of the subject, and that few studies have been conducted in this area, it is recommended that the present study should be conducted in different countries to develop nursing knowledge. Also, nursing managers can use the results of this study to improve the quality patient care and safe nursing care in clinical nurses. Accordingly, the present study was conducted to investigate the relationship of moral courage, teamwork, and safe nursing care in clinical nurses.

## Methods

### Study design

The present descriptive cross-sectional multicenter study was carried out from December 2023 to February 2024, in three educational hospitals (Vali Asr, Shariati, and Imam Hossein) affiliated with the Fasa University of Medical Sciences, in Fars Province, Southern Iran.

### Participants

We calculated the sample size using the formula used in a previous study (β = 0.2, α = 0.05, correlation coefficient: *r* = 0.27) [23]. The sample size was estimated 250 nurses. Due to the possibility of incomplete completion of questionnaires by the study participants, we considered more samples. A total of 400 nurses working at three educational hospitals affiliated with the Fasa University of Medical Sciences in the south of Fars province were selected using convenience sampling. In the present study, 375 participants filled out and returned the questionnaires. Thus, the response rate was 93.75%.

$${\varvec{u}}=\mathbf{1}/\mathbf{2}\mathbf{l}\mathbf{n}\frac{(\mathbf{1}+{\varvec{r}})}{(\mathbf{1}-{\varvec{r}})}$$

$${\varvec{n}}=\frac{({{\varvec{Z}}}_{\boldsymbol{1-\alpha /2}}+{{\varvec{Z}}}_{\mathbf{1-}{\varvec{\beta}}}{)}^{2}}{{{\varvec{u}}}^{\mathbf{2}}}+3$$

The inclusion criteria considered in this study were being willing to participate in the study, having at least one year of work experience, and signing informed consent to participate in the study. The exclusion criteria were incomplete questionnaires and unwillingness to continue cooperation with the study for any reason.

### Data collection method

The first author (ZA) visited the nursing offices of each of the three hospital and obtained permission to conduct the research. A list was obtained of all departments where nurses were clinically active, along with the number and names of nurses employed in those departments. Nurses were selected from each department based on the inclusion criteria. The researcher visited each department at the beginning of each shift, explained the research, and obtained written informed consent from eligible nurses with the questionnaires collected at the end of the shift.

### Data collection instruments

Data were collected using A demographic survey, Nurses’ Moral Courage Questionnaire (NMCQ), Team STEPPS Team Perception Questionnaire (T-TPQ), and the Assessment of Safe Nursing Care Questionnaire (ASNCQ) were the instruments used to collect the data in this study.

### Demographic survey

The demographic checklist included questions on age, gender, marital status, level of education, place of work, and clinical work experience.

### Nurses’ Moral Courage Questionnaire (NMCQ)

Sadooghi et al. in 2016 designed and validated The NMCQ [24]. This questionnaire contains 20 items and evaluates three dimensions: moral self-fulfillment (9 questions), risk‐taking (8 questions), and the ability to defend the right (3 questions); the items are scored using a 5-point Likert scale ranging from always (score 5) to never (score 1) in a self-report manner. The weight of the items varies from 3 to 7 and the score of each item is obtained through multiplying the Likert score by the value of the item. The minimum score of this questionnaire is 102 and its maximum score is 510. In this questionnaire, the scores 102–238 indicate low moral courage, 239–374 medium moral courage, and 375–510 high moral courage. The content validity index (CVI) of the questionnaire was obtained 0.87, its internal consistency was 0.88 using Cronbach's alpha coefficient, and its consistency was 0.87 using the test–re-test and calculating the intra-class correlation coefficient [24]. In this study, the Cronbach’s α value was 0.89, which is considered a high level of reliability for the scale.

### Team STEPPS Team Perception Questionnaire (T-TPQ)

Battles together with King developed and validated this questionnaire in 2010 [25]. It contains 35 items with five subscales of “team structure”, “team leadership”, “situational monitoring”, “mutual support”, and “communication”. Each subscale comprises seven items, and they are scored using a 5-point Likert scale ranging from “Strongly disagree = 1” to “Strongly agree = 5.” The total score is calculated by summing the ratings for each item (35 items) and ranges from 35 to 175. The scores 35–81 indicate low teamwork, 82–128 medium teamwork, and 129–175 high teamwork. Kakemam et al. translated and validated the questionnaire in Iran in 2021. Cronbach’s alpha coefficient of the Persian version of this questionnaire was 0.96 [26]. The Cronbach’s α value in the present study was 0.92.

### Assessment of Safe Nursing Care Questionnaire (ASNCQ)

Rashvand et al. designed and validated the ASNCQ [27]. It evaluates safe nursing care in the context of the Iranian healthcare system. This questionnaire contains 32 questions divided into four sections. The first part evaluates the nursing skills (16 questions), the second part the patient's psychological needs (4 questions), the third part the patient's physical needs (7 questions), and the fourth part the nurse's teamwork (5 questions). The items are scored using a 5-point Likert scale ranging from never (1 point) to forever (5 points). The weight of questions 14, 18, 19, 20, and 32 is equal to 1; that of questions 2, 3, 4, 5, 7, 10, 11, 12, 13, 15, 16, 17, 21, 26, and 30 is 2. Further, the weight of questions 1, 6, 8, 9, 23, 24, 25, 27, 29, and 31 is 3, and that of questions 28 and 22 is 4. Thus, the obtained number was multiplied by the weight of the question, and the final number was used for the analysis. Scores 73 to 170 indicate poor performance, 171 to 267 moderate performance, and a score of 268 to 365 good performance. The reliability of this tool using Alpha Cronbach's method was 0.97, indicating its good reliability. In this study, Cronbach’s α value was 0.95, showing a high level of reliability for the scale.

### Data analysis

The data were analyzed using SPSS version 22.0. The total scores for moral courage, team work, and safe care, as well as the scores for each dimension, are shown as mean and standard deviation. We also used Chi-square test, independent t-test, and Pearson’s correlation coefficients to find out the association of moral courage, teamwork, safe nursing care, and demographic variables among nurses. *P* values < 0.05 were considered as the level of significance. Finally, the demographic features, moral courage, and teamwork, found to correlate with safe nursing care (*P* < 0.05), were entered into multivariate linear regression with the backward technique.

### Ethical considerations

All the participants gave written informed consent to participate in the study. This study was conducted based on the principles of the revised Declaration of Helsinki, which is a statement of ethical principles used to guide medical researchers who investigate human subjects. The participants were assured about the anonymity and confidentiality of their information. Furthermore, this study was approved by the Institutional Research Ethics Committee of Fasa University of Medical Sciences, Fasa, Iran (ethical code: IR.FUMS.REC.1402.157).

## Results

Of the 375 eligible nurses who participated in this study, 250 (66.7%) were female. The mean ± SD of the participants’ age was 32.66 ± 6.63 years, and that of their work experience was 8.56 ± 6.22 years. The participants’ demographic characteristics are reported in Table 1.

**Table 1:** The demographic characteristics of participants (*N* = 375)

		Frequency	Percent
Gender	Male	125	33.3%
	Female	250	66.7%
Marital status	Single	127	33.9%
	Married	248	66.1%
Age	< 40	311	82.9%
	≥ 40	64	17.1%
Work experience	≤ 5	158	42.1%
	> 5	217	57.9%
Education	B.Sc	342	91.2%
	M.Sc	33	8.8%
Place of work	General	184	49.1%
	Special care units	113	30.1%
	Pediatric	41	10.9%
	Emergency	37	9.9%
		Mean	SD
Age (year)		32.66	6.63
Work experience (year)		8.56	6.22

*SD* Standard deviation, *B.Sc.* Bachelor of Science, *M.Sc.* Master of Science

### Descriptive statistics of moral courage, teamwork, and safe care by clinical nurses

In this study, the nurses revealed to have high levels of moral courage, teamwork, and safe nursing care, as outlined in Table 2. The total mean score ± SD of the nurses’ moral courage was found to be 422.37 ± 52.92, showing its high level among participants. The total mean score ± SD of their teamwork was 144.09 ± 18.43, indicating an acceptable and high level of teamwork. Also, the total mean score ± SD of their safe nursing care scores was 315.84 ± 41.95, which indicates good performance.

**Table 2:** The scores of moral courage, team work, and safe nursing care

Variable domains			Minimum	Maximum	Mean	SD
Moral courage	Moral self-fulfillment	55–275	127	275	229.43	36.50
	Risk-taking	36–180	72	180	148.21	26.56
	Ability to defend the right	11–55	23	55	44.73	7.74
	Total score	102–510	243	510	422.37	52.92
Team work	Team structure	7–35	18	35	29.58	3.42
	Team leadership	7–35	11	35	29.32	5.88
	Situational monitoring	7–35	17	35	28.35	3.68
	Moral support	7–35	18	35	28.06	4.24
	Communication	7–35	13	35	28.77	4.65
	Total score	35–175	94	173	144.09	18.43
Safe care	Assessment of Nursing skills	35–175	98	175	148.04	20.72
	Evaluation of psychological safety	5–25	15	25	22.43	3.02
	Evaluation of physical safety	20–100	56	100	89.37	11.86
	Evaluation of teamwork skills	13–65	26	65	56.00	10.06
	Total score	73–365	211	365	315.84	41.95

*SD* Standard deviation

### Correlation analysis of moral courage, teamwork, and safe care by nurses

A statistically significant positive correlation was found between teamwork and safe care (*r* = 0.57, *p* < 0.001), teamwork and moral courage (*r* = 0.49, *p* = 0.002), and moral courage and safe nursing care (*r* = 0.59 *p* < 0.001) (Table 3).

**Table 3:** Correlations of moral courage, teamwork, and safe nursing care

	Team work		Safe care
	r	*P*-value	r *P*-value
Moral courage	0.49	0.002	0.59 *p* < 0.001
Team work			0.57 *p* < 0.001

r: Pearson correlation coefficient

### Correlation of moral courage, teamwork, and safe care in demographic variables

Table 4 presents the results of comparison of moral courage, teamwork, and safe care in demographic variables. The results revealed moral courage correlated with age and work experience (*p* < 0.05). Further, teamwork and safe nursing care correlated with work experience (*p* < 0.05).

**Table 4:** Correlations of moral courage, teamwork, and safe care in demographic variables

		Moral courage			Team work			Safe care		
		Mean	SD	*p*-value	Mean	SD	*p*-value	Mean	SD	*p*-value
Gender	Male	421.99	51.02		142.87	19.38		318.09	38.82	
	Female	422.56	53.95	0.923	144.69	17.94	0.381	314.71	43.46	0.463
marital status	Single	423.62	53.72		146.31	17.09		314.17	40.73	
	Married	421.73	52.61	0.743	142.95	19.01	0.395	316.69	42.62	0.583
Education	B.Sc	421.31	53.37		144.25	18.32		315.62	42.32	
	M.Sc	433.36	47.41	0.212	142.36	19.78	0.575	318.12	38.47	0.725
Place of work	General	420.05	54.41		146.66	17.93		316.34	41.02	
	Special care units	420.70	50.30		141.96	19.71		320.33	42.02	
	Pediatric	433.00	54.37	0.489	141.41	17.69	0.565	306.46	41.70	0.253
	Emergency	427.22	52.05		140.73	16.45		310.00	45.90	
		Age					Work experience			
		r		*p*-value			r			*p*-value
Moral courage		0.012		0.024			0. 24			0.078
Team work		0.001		0.996			0.37			0.002
Safe care		0.027		0.607			0.47			0.008

*r* Pearson correlation coefficient

### Predictive factors of the demographic characteristics, moral courage, and teamwork on safe nursing care

Table 5 indicates the results of multiple linear regression analysis regarding the predictive role of demographic characteristics, moral courage, and teamwork in safe nursing care. The results showed that age, work experience, moral courage, and teamwork explained 44.4% of the variance in safe nursing care (R2 = 0.44, *p* < 0.001). In addition, work experience (β = 9.963, *p* = 0.027), moral courage (β = 0.082, *p* = 0.009), and teamwork (β = 5.858, *p* < 0.001) had the highest predictive impact on safe nursing care. In other words, a one‑unit increase in the score of teamwork enhanced the safe nursing care by 5.85 units.

**Table 5:** Factors predicting nurse’s safe care (Adjusted R square = 0.44)

	B	SE of B	Beta	t	*p*-value
Model constant	104.031	30.138		3.062	0.002
Age (year)	0.348	1.144	0.055	0.304	0.061
Work experience (year)	9.963	4.473	-0.113	2.227	0.027
Gender	-3.568	4.118	-0.040	-0.867	0.387
Marital status	-.940	1.199	0.113	-.0.784	0.433
Education	-1.445	5.919	-0.010	-0.244	0.807
Special care units	7.325	3.807	0.080	1.924	0.055
Pediatric	-4.036	5.541	-0.030	-0.729	0.467
Emergency	-4.298	5.757	-0.031	-0.747	0.456
Moral courage	0.082	0.031	0.103	2.624	0.009
Team work	5.858	0.356	0.649	16.463	< 0.001

*B* Regression coefficient, *SE of B* Standard error of B, *Beta* Standardized beta coefficient

## Discussion

This study aimed to explore the relationship between moral courage, teamwork, and safe nursing care in clinical nurses from December 2023 to February 2024 in three hospitals in the south of Iran. In the present study, the level of moral courage among nurses was high. In line with the findings of this study, some studies have revealed that nurses have reported a high moral courage [9, 28]. However, other studies reported the moral courage of nurses to be moderate and low [29, 30]. This difference might be attributed to the participants’ different ethical climate, organizational and cultural contexts, work experience, inappropriate working conditions and work environment, lack of organizational support as well as lack of job motivation [31, 32]. However, the findings of study, Numminen, et al., showed that the individual and organizational factors, such as positive personal experiences, commitment to ethical principles, supportive work environment, and teamwork, were associated with moral courage in nursing [33]. The findings of this study also indicate that the individual factors including work experience and age are directly related to the level of moral courage of nurses participating in this study.

According to the studies of Abdollahi and Goktas, a positive correlation exists between age and years of nursing experience, which in turn, appears to influence the development of moral courage in nurses. This suggests that as nurses age and accumulate experience, they encounter a wider range of ethical challenges. Consequently, their ability to navigate these situations ethically may be enhanced thanks to their increased knowledge and prior experiences in similar circumstances [34, 35].

The score of nurses' teamwork in this study was high. In line with this study, Nobahar, et al. in Iran revealed that teamwork among nurses was high [19]. The studies carried out by Champman, et al. in Australia, Goh, et al. in Singapore as well as, Baek, et al. in Korean reported that teamwork was high among nurses [36–38].

However, the studies carried out by Li in Taiwan and Hwang et al. in Korea reported that teamwork was rated as moderate [39, 40]. The reason for this discrepancy in the results might be the organizational climate differences between nurses in different countries. However, the findings of the study conducted by Behnia, et al., aimed at investigating the affecting factors of teamwork among nurses in Iran, demonstrated that factors such as such as training in teamwork skills, job satisfaction, increased workload and staff shortages, cooperation and mutual understanding among members of the healthcare team regarding their respective roles were found to exert a substantial influence on teamwork [41].

Based on the findings of our study, safe nursing care was at a high level among nurses. Consistent with the findings of this study, Khodaveisi, et al. as well as Mohammadi et al. also noted that the safe nursing care was at a high level among nurses [7, 23]. Further, the results of the present study indicated a strong and direct correlation among the participants' moral courage, teamwork, and safe care nursing. In line with the findings of this study, Khodaveisi, et al. found that moral courage correlated with safe nursing care in nurses who cared for COVID-19 patients. The reason could be the nurses' sense of commitment to a useful and constructive presence to save the patient's lives and take care of humanity in this global crisis [7]. Also, the results of another study conducted by Mohammadi et al. revealed that moral courage and moral sensitivity significantly correlated with safe care [23]. However, the results of another study conducted by Kashani, et al. showed that there is a positive and significant relationship between safety care and moral courage [42].

The results of this study revealed that work experience was a positive predictor of teamwork, moral courage, and safe nursing care. However, Nobahar, et al. found that the ICU nurses’ work experience was a positive predictor of teamwork and a negative predictor of missed nursing care. Obviously, caring for a patient with complicated critical care needs requires stronger teamwork [19]. Other studies in line with the findings of this study noted that increasing work experience augments the moral courage in nurses, and then they show more courage in performing care. This relationship is probably due to increase in an individual's awareness of organizational conditions, gaining professional and practical competence, and learning courageous behaviors from other colleagues [43, 44].

### Strengths

To the best of our knowledge, this is the first study in Iran carried out to determine how moral courage and teamwork correlate with safe nursing care in clinical nurses, which is a novelty. Also, one of the strengths of this study has been the use of comprehensive and specific questionnaires for assessing moral courage, teamwork, and safe nursing care.

### Limitations

One of the limitations was that data collection was done through a self‑report questionnaire and the participants’ responses may not have been truthful, so bias could have been present. In the present study, only nurses participated; it is recommended that the study be conducted on nursing students as well. Meanwhile, in view of the economic, cultural, and social differences between Iran and other countries, it is recommended that similar studies should also be conducted in other countries.

## Conclusion

The results of this study indicated that the nurses’ moral courage and teamwork were positively and significantly correlated with their safe nursing care. Accordingly, it is recommended that nursing managers be conscious of the role of moral courage, and team work in the nursing workplace in enhancing safe nursing care.

## Data Availability

The data that support the findings of this study are available from the corresponding author upon reasonable request.
